# METTL3介导m6A修饰长链非编码RNA THAP7-AS1表达上调促进肺癌发生的作用及机制研究

**DOI:** 10.3779/j.issn.1009-3419.2023.102.45

**Published:** 2023-12-20

**Authors:** Yu ZHANG, Yanhong WANG, Mei LIU

**Affiliations:** ^1^730900 白银，甘肃省白银市第二人民医院病理科（张瑜，王彦宏）; ^1^Department of Pathology, The Second People's Hospital of Baiyin City; ^2^中国人民解放军68303部队卫生连（刘美）; ^2^Health Company of PLA 68303 Troops, Baiyin 730900, China

**Keywords:** 甲基转移酶样3, N6-甲基腺苷, 长链非编码RNA THAP7-AS1, 肺肿瘤, 增殖, Methyltransferase-like 3, N6-methyladenosine, Long non-coding RNA THAP7-AS1, Lung neoplasms, Proliferation

## Abstract

**背景与目的** 肺癌是对人类健康的一大威胁，有关肺癌发生发展的分子机制复杂且知之尚少，探索与肺癌发展相关的分子标志物有利于提高早期诊断和治疗的效果。长链非编码RNA（long non-coding RNA, lncRNA）THAP7-AS1已知在胃癌中高表达，但在其他癌症中研究较少。本研究旨在探究甲基转移酶样3（methyltransferase-like 3, METTL3）介导N6-甲基腺苷（N6-methyladenosine, m6A）修饰lncRNA THAP7-AS1表达上调促进肺癌发生的作用及机制。**方法** 收集120例肺癌与对应癌旁组织样本，lncRNA微阵列分析差异表达的lncRNA，实时荧光定量聚合酶链式反应（real-time quantitative polymerase chain reaction, qRT-PCR）检测肺癌、癌旁组织、肺癌细胞系THAP7-AS1表达，分析THAP7-AS1对肺癌的诊断价值以及其表达水平与肺癌患者生存率、临床病理特征的关系。通过生物信息学分析、甲基化RNA免疫共沉淀（methylated RNA immunoprecipitation, meRIP）、RNA pull-down实验、RIP实验探究THAP7-AS1的分子调节机制；通过MTS、克隆形成、划痕、Transwell、体内异种移植实验测定各组SPC-A-1、NCI-H1299细胞增殖、迁移、侵袭、成瘤能力，Western blot检测磷脂酰肌醇-3激酶/蛋白激酶B（phosphoinositide 3-kinase/protein kenase B, PI3K/AKT）信号通路蛋白表达。**结果** 肺癌组织、细胞系THAP7-AS1表达升高（P<0.05），对肺癌具有一定的诊断价值[曲线下面积（area under the curve, AUC）=0.737]，其表达水平与患者总生存率、肿瘤大小、肿瘤原发灶-淋巴结-转移（tumor-node-metastasis, TNM）分期、淋巴结转移相关（P<0.05）。METTL3介导的m6A修饰能够增强THAP7-AS1表达。与SPC-A-1、NCI-H1299细胞NC组、sh-NC组相比，THAP7-AS1组增殖、迁移、侵袭能力提高（P<0.05），移植瘤体积、质量增大（P<0.05），sh-THAP7-AS1组增殖、迁移、侵袭能力下降（P<0.05）。THAP7-AS1与Cullin蛋白4B（Cullin 4B, CUL4B）存在特异结合。与SPC-A-1、NCI-H1299细胞Vector组相比，THAP7-AS1组增殖、迁移、侵袭能力、磷脂酰肌醇-4,5-二磷酸3-激酶催化亚基α（phosphatidylinositol-4,5-bisphosphate 3-kinase catalytic subunit alpha, PI3KCA）、磷脂酰肌醇-3激酶催化亚基δ（phosphoinositide-3 kinase-catalytic subunit delta, PI3KCD）、磷酸化磷脂酰肌醇3-激酶（phospho-phosphatidylinositol 3-kinase, p-PI3K）、磷酸蛋白激酶B（phospho-protein kinase B, p-AKT）、磷酸哺乳动物雷帕霉素靶蛋白（phospho-mammalian target of rapamycin, p-mTOR）表达水平升高（P<0.05）。**结论** LncRNA THAP7-AS1通过METTL3介导的m6A修饰稳定表达，与CUL4B结合激活PI3K/AKT信号通路，促进肺癌发生发展。

## Abstract (en)

**Background and objective** Lung cancer is a major threat to human health. The molecular mechanisms related to the occurrence and development of lung cancer are complex and poorly known. Exploring molecular markers related to the development of lung cancer is helpful to improve the effect of early diagnosis and treatment. Long non-coding RNA (lncRNA) THAP7-AS1 is known to be highly expressed in gastric cancer, but has been less studied in other cancers. The aim of the study is to explore the role and mechanism of methyltransferase-like 3 (METTL3) mediated up-regulation of N6-methyladenosine (m6A) modified lncRNA THAP7-AS1 expression in promoting the development of lung cancer. **Methods** Samples of 120 lung cancer and corresponding paracancerous tissues were collected. LncRNA microarrays were used to analyze differentially expressed lncRNAs. THAP7-AS1 levels were detected in lung cancer, adjacent normal tissues and lung cancer cell lines by quantitative reverse transcription-polymerase chain reaction (qRT-PCR). The diagnostic value of THAP7-AS1 in lung cancer and the relationship between THAP7-AS1 expression and survival rate and clinicopathological parameters were analyzed. Bioinformatics analysis, methylated RNA immunoprecipitation (meRIP), RNA pull-down and RNA-immunoprecipitation (RIP) assay were used to investigate the molecular regulation mechanism of THAP7-AS1. Cell proliferation, migration, invasion and tumorigenesis of SPC-A-1 and NCI-H1299 cells were determined by MTS, colony-formation, scratch, Transwell and xenotransplantation in vivo, respectively. Expression levels of phosphoinositide 3-kinase/protein kenase B (PI3K/AKT) signal pathway related protein were detected by Western blot. **Results** Expression levels of THAP7-AS1 were higher in lung cancer tissues and cell lines (P<0.05). THAP7-AS1 has certain diagnostic value in lung cancer [area under the curve (AUC)=0.737], and its expression associated with overall survival rate, tumor size, tumor-node-metastasis (TNM) stage and lymph node metastasis (P<0.05). METTL3-mediated m6A modification enhanced THAP7-AS1 expression. The cell proliferation, migration, invasion and the volume and mass of transplanted tumor were all higher in the THAP7-AS1 group compared with the NC group and sh-NC group of SPC-A-1 and NCI-H1299 cells, while the cell proliferation, migration and invasion were lower in the sh-THAP7-AS1 group (P<0.05). THAP7-AS1 binds specifically to Cullin 4B (CUL4B). The cell proliferation, migration, invasion, and expression levels of phosphatidylinositol-4,5-bisphosphate 3-kinase catalytic subunit alpha (PIK3CA), phosphoinositide-3 kinase, catalytic subunit delta (PIK3CD), phospho-phosphatidylinositol 3-kinase (p-PI3K), phospho-protein kinase B (p-AKT) and phospho-mammalian target of rapamycin (p-mTOR) were higher in the THAP7-AS1 group compared with the Vector group of SPC-A-1 and NCI-H1299 cells (P<0.05). **Conclusion** LncRNA THAP7-AS1 is stably expressed through m6A modification mediated by METTL3, and combines with CUL4B to activate PI3K/AKT signal pathway, which promotes the occurrence and development of lung cancer.

肺癌是癌症致死的主要原因之一^[1]^，尽管肺癌的早期诊断与治疗等方面已取得了许多进展，但仍缺乏有效的早期检测和预后监测的生物标志物，患者5年生存率低，仅为21%^[2,3]^。长链非编码RNA（long non-coding RNA, lncRNA）可通过多种机制调节基因表达，进而影响细胞增殖、迁移、代谢等方面^[4]^。N6-甲基腺苷（N6-methyladenosine, m6A）修饰是真核细胞中最常见的内部RNA修饰机制，参与调节RNA剪接、稳定、降解、翻译等机制，是一个高度动态、可逆的过程，此过程发生异常可导致下游基因表达失调，影响细胞功能^[5,6]^。m6A修饰受“写入”“读取”“擦除”3个同源因子的控制，“写入”因子是将甲基添加到m6A修饰位点的m6A甲基转移酶，包括甲基转移酶样（methyltransferase-like, METTL）3、METTL14、METTL16和Wilms肿瘤相关蛋白等^[7,8]^，近年来多项研究^[9⇓-11]^显示，m6A甲基化修饰异常在血液系统疾病、中枢神经系统疾病、生殖系统疾病以及癌症等多种疾病中发挥重要作用。LncRNA THAP7-AS1是一个天然的反义lncRNA，位于染色体22q11.21，其表达在胃癌中显著上调^[12]^，目前关于lncRNA THAP7-AS1在肺癌中的作用尚不清楚。本研究通过生物信息学分析与体内外实验探究METTL3介导m6A修饰lncRNA THAP7-AS1影响肺癌进展的作用机制，为肺癌发展的分子机制提供新的理论依据。

## 1 资料与方法

### 1.1 组织标本

收集2021年1月至2023年1月经手术切除的120例肺癌组织及相应癌旁样本，术后病理诊断确诊为肺癌^[13]^。其中男性68例，女性52例，年龄44-76（59.74±11.53）岁。研究已获得甘肃省白银市第二人民医院医学伦理委员会批准，患者均知情并自愿提供组织样本。

### 1.2 实验动物

20只SPF级4周龄健康雄性BALB/c裸鼠，体重（30±5）g，购自河南省实验动物中心，动物合格证号SCXK（豫）2022-0001。适应性饲养1周后进行实验，实验操作均符合3R原则，动物实验伦理审批号：GSCM-20210112。

### 1.3 实验细胞

SPC-A-1（货号：FS-X1751）、NCI-H1299（货号：FS-X1698）、LTEP-a-2（货号：FS-X1679）、H460（货号：A01X890），购自上海抚生实业有限公司；人肺癌细胞A549（货号：BNCC337696），购自北京北纳创联生物技术研究院；人正常肺支气管上皮细胞BEAS-2B（货号：SNL-203），购自武汉尚恩生物技术有限公司；人肾上皮细胞293T（货号：HEK293T），购自上海赛百慷生物技术股份有限公司。

### 1.4 主要试剂与仪器

DMEM培养基（货号：11965092）、胎牛血清（货号：16140063）、链霉素/青霉素（货号：15070063）、TRIzol试剂（货号：15596018）、High-Capacity cDNA反转录试剂盒（货号：4368813）、链霉素亲和偶联磁珠（货号：88817），购自美国Thermo Fisher公司；慢病毒表达载体的构建、鉴定、包装与滴度测定由上海美轩生物科技有限公司负责完成；SYBR Premix Ex Taq II试剂盒（货号：RR820A），购自大连宝生生物科技有限公司；聚合酶链式反应（polymerase chain reaction, PCR）引物与内参购自武汉金开瑞生物工程有限公司；荧光原位杂交（fluorescence in situ hybridization, FISH）试剂盒（货号：Bes1001），购自广州伯信生物科技有限公司；MTS试剂盒（货号：15711），购自西安百萤生物科技有限公司；5-乙炔基-2'脱氧尿嘧啶核苷（5-ethynyl-2′-deoxyuridine, EdU）细胞增殖检测试剂盒（货号：A003-A008），购自美国GeneCopoeia公司；Matrigel基质胶（货号：354234），购自上海研卉生物科技有限公司；IgG（货号：ab172730）、m6A（货号：ab286164）、信号转导与转录活化因子3（signal transducers and activators of transcription 3, STAT3）（1:1000，货号：ab68153）、Cullin蛋白4B（CUL4B，1:500，货号：ab227724）、大核糖体蛋白P0（ribosomal protein large P0, RPLP0）（1:1000，货号：ab192866）、磷脂酰肌醇-4,5-二磷酸3-激酶催化亚基α（phosphatidylinositol-4,5-bisphosphate 3-kinase catalytic subunit alpha, PI3KCA）（1:1000，货号：ab40776）、磷脂酰肌醇-3激酶催化亚基δ（phosphoinositide-3 kinase catalytic subunit delta, PI3KCD）（1:1000，货号：ab109006）、磷酸化磷脂酰肌醇3-激酶（phospho-phosphatidylinositol 3-kinase, p-PI3K）（1:200，货号：ab182651），购自英国Abcam公司；磷酸化蛋白激酶B（phospho-protein kinase B, p-AKT）（1:2000，货号：4060）、磷酸化哺乳动物雷帕霉素靶蛋白（phospho-mammalian target of rapamycin, p-mTOR）（1:1000，货号：5536），购自美国CST公司。其他试剂均为市售分析纯。

罗氏LightCycler480 II PCR仪购自源场芯科技设备（上海）有限公司；Leica DM4B光学显微镜、徕卡DM1000生物荧光显微镜购自德国Leica公司；LF-Mini4型小型垂直电泳槽购自北京龙方科技有限公司。

### 1.5 实验方法

#### 1.5.1 细胞培养、分组与转染

复苏SPC-A-1、LTEP-a-2、A549、NCI-H1299、H460和BEAS-2B细胞，使用含10%胎牛血清与100 mg/mL链霉素/青霉素的DMEM培养基，于37 °C、5% CO_2_、饱和湿度的电热恒温培养箱中进行培养。SPC-A-1、NCI-H1299细胞分为NC组（转染NC）、sh-NC组（转染sh-NC）、METTL3组（转染METTL3）、sh-METTL3组（转染sh-METTL3）、THAP7-AS1组（转染THAP7-AS1）、sh-THAP7-AS1组（转染sh-THAP7-AS1）、Vector组（转染NC+sh-NC）和THAP7-AS1+sh-CUL4B组（转染THAP7-AS1+sh-CUL4B），按照分组名称进行慢病毒转染，构建稳定表达的细胞株。

#### 1.5.2 实时荧光定量PCR（quantitative reverse transcription-PCR, qRT-PCR）检测lncRNA THAP7-AS1相对表达情况

使用TRIzol试剂提取各组组织、细胞总RNA后用反转录试剂盒反转录成cDNA。使用1 μg cDNA模板、上下游引物各0.5 μg和SYBR Premix Ex Taq II试剂盒配制20 μL PCR反应体系，扩增条件：95 °C预变性2 min，95 °C 15 s，60 °C 60 s，循环40次。表1为引物序列，应用2^-△△CT^法计算THAP7-AS1的相对水平。

**表1:** 引物序列

Primers	Sequence (5'-3')
THAP7-AS1	F: CGGCTAGCCTGTGGAGCCACAAACCCGTGAGCA
	R: CCCAAGCTTTCTTTCCCAGATGCCGCGTCACTGC
β-actin	F: GCTCTCTTCCAGCCTTCCTTCCTG
	R: GTGTTGGCGTACAGGTCCTTGCGG

#### 1.5.3 FISH实验

SPC-A-1、NCI-H1299细胞常规接种于24孔板内的载玻片上，培养24 h后加入4%多聚甲醛固定10 min。加入0.5% Triton X-100通透处理5 min。预杂交液、杂交液37 °C预热30 min后加至载玻片上，置于37 °C环境中反应20 min。杂交液1:50稀释THAP7-AS1探针，加至载玻片上，37 °C避光过夜。洗脱未结合的探针，加入4',6-二脒基-2-苯基吲哚（4',6-diamidino-2-phenylindole, DAPI）避光反应8 min，于荧光显微镜下拍照。

#### 1.5.4 甲基化RNA免疫共沉淀（methylated RNA immunoprecipitation, meRIP）实验

取SPC-A-1、NCI-H1299细胞总RNA，以IgG抗体为对照，目的抗体为m6A，4 °C孵育过夜。加入蛋白G琼脂糖珠，4 °C孵育2 h，加入20 mmol/L N6-甲基腺苷5'-单磷酸酯钠盐，4 °C洗脱2次，1 h后经RNA纯化试剂盒纯化，qRT-PCR分析m6A富集情况。

#### 1.5.5 MTS法检测细胞增殖能力

各组转染SPC-A-1、NCI-H1299细胞接种至96孔板上，接种密度2000个/孔，于培养24、48、72 h后进行MTS检测，每孔加入40 μL MTS试剂，避光培养4 h，于490 nm波长下检测各孔吸光度。

#### 1.5.6 EdU检测DNA复制活性

SPC-A-1、NCI-H1299 NC组、THAP7-AS1组、sh-NC组、sh-THAP7-AS1组细胞以4000个/孔的接种密度接种于24孔板中，37 °C、5% CO_2_培养箱中培养12 h。加入含EdU工作液的培养基培养2 h，4%多聚甲醛固定后加入Triton X-100通透细胞，加入Click反应液，37 °C避光反应30 min，加入DAPI染核，37 °C避光反应10 min，于荧光显微镜下拍照，计算EdU阳性细胞的百分比。

#### 1.5.7 克隆形成实验检测细胞克隆形成能力

SPC-A-1、NCI-H1299 NC组、THAP7-AS1组、sh-NC组、sh-THAP7-AS1组细胞接种至6孔板中，接种密度2000个/孔，培养10 d。弃去培养基，加入甲醇固定30 min，1%结晶紫染色20 min，统计克隆数量。

#### 1.5.8 划痕实验检测细胞迁移能力

各组转染SPC-A-1、NCI-H1299细胞接种至6孔板中，接种密度1×10^6^个/孔，观察细胞覆盖率达到90%，用移液器枪头作划痕，清除脱落的细胞，加入无血清培养基继续培养24 h。于划痕后（0 h）和培养24 h时拍照，测量划痕宽度，计算细胞迁移率=（0 h宽度-24 h宽度）/0 h宽度×100%。

#### 1.5.9 Transwell实验检测细胞侵袭能力

无血清DMEM/F12培养基与Matrigel基质胶按1:3的比例稀释，混匀后均匀涂抹于小室上室膜底部，置于培养箱中过夜，次日置于紫外线灯下照射30 min。各组转染SPC-A-1、NCI-H1299细胞制成细胞悬液，接种于Transwell上室，下室加入含10%胎牛血清的DMEM培养基，正常培养24 h。4%多聚甲醛固定进入下室的细胞，结晶紫染色，于光镜下观察拍照，计算细胞侵袭率=各组侵袭细胞数/对照组侵袭细胞数×100%。

#### 1.5.10 体内异种移植实验

20只裸鼠根据不同肿瘤细胞平均分为两部分，每部分根据细胞分组名称分为NC组、THAP7-AS1组，每组5只。于左侧腹部皮下接种各组细胞，细胞悬液密度为2×10^6^个/mL。从接种后1 d起，每4 d测量一次肿瘤体积，计算公式：体积=1/2（长×宽^2^）。21 d后观察到接种位置出现明显肿块，完整取出肿瘤并称重。

#### 1.5.11 RNA pull-down实验

SPC-A-1、NCI-H1299细胞加入适量预冷细胞裂解液，充分裂解后于4 °C、10,000 rpm条件下离心10 min，上清液即为总蛋白。体外转录生物素标记的THAP7-AS1和反义链RNA，经纯化后分别加入SPC-A-1、NCI-H1299细胞总蛋白与链霉亲和素磁珠，4 °C孵育过夜后离心收集磁珠。RNA-蛋白质复合物经洗脱、变性、SDS-PAGE凝胶电泳后进行银染，切取THAP7-AS1与反义链RNA之间的差异条带，经消化后进行质谱分析。

#### 1.5.12 RIP实验

使用细胞裂解液裂解SPC-A-1、NCI-H1299细胞，分别加入IgG与CUL4B抗体偶联的磁珠，于4 °C环境下孵育过夜，加入0.1%十二烷基硫酸钠和0.5 mg/mL蛋白酶K，于55 °C环境中孵育30 min去除蛋白质。测定RNA浓度与纯度后进行qRT-PCR检测THAP7-AS1的相对表达水平。

#### 1.5.13 Western blot检测磷脂酰肌醇-3激酶/蛋白激酶B（phosphoinositide 3-kinase/protein kinase B, PI3K/AKT）信号通路相关蛋白表达情况

收集SPC-A-1、NCI-H1299 Vector组、THAP7-AS1组、THAP7-AS1+sh-CUL4B组细胞，加入适量裂解液裂解后4 °C、10,000 rpm离心10 min，保留上清液，BCA定量后制样。经凝胶电泳、转膜后截取目的条带，加入封闭液室温封闭1 h。加入稀释后的对应一抗孵育液：STAT3（1:1000）、CUL4B（1:500）、RPLP0（1:1000）、PI3KCA（1:1000）、PI3KCD（1:1000）、p-PI3K（1:200）、p-AKT（1:2000）、p-mTOR（1:1000），4 °C孵育过夜。洗膜，加入1:5000稀释的对应二抗孵育液，室温孵育2 h，洗膜，加入ECL试剂，避光反应5 min后收集荧光成像结果。

#### 1.5.14 统计学方法

数据分析使用SPSS 24.0软件完成，计量资料使用均数±标准差（Mean±SD）表示，两组间比较采用t检验，多组间比较采用单因素方差分析，计数资料以率（%）表示，分类变量比较采用χ^2^检验，两变量间相关性采用Spearman相关性分析。使用受试者工作特征（receiver operating characteristic, ROC）曲线分析THAP7-AS1对肺癌的诊断价值，计算ROC曲线下面积（area under the curve, AUC）及其95%CI。使用Kaplan-Meier Plotter数据库（http://kmplot.com/analysis/）分析不同THAP7-AS1表达水平的肺癌患者的生存情况。P<0.05为差异有统计学意义。

## 2 结果

### 2.1 THAP7-AS1在肺癌中表达上调

从本研究收集到的组织中随机选择了10例肺癌与2例癌旁组织进行lncRNA微阵列分析，结果显示，肺癌组织中有425个lncRNA显著下调，989个lncRNA显著上调（折叠变化>2.0、P<0.05）。根据折叠变化≥5、P<0.05、与癌旁组织相比表达水平明显上调3个条件进一步筛选出LOC100133669与THAP7-AS1两个候选lncRNA。qRT-PCR检测全部120对肺癌与癌旁组织，结果显示肺癌组织中THAP7-AS1水平较癌旁组织显著上调（P<0.05），因此本研究着重探索THAP7-AS1在肺癌发生发展中的作用。与正常支气管上皮细胞BEAS-2B相比，SPC-A-1、LTEP-a-2、A549、NCI-H1299、H460细胞THAP7-AS1表达量均上调（P<0.05），SPC-A-1与NCI-H1299细胞中THAP7-AS1 mRNA水平最高，因此选择SPC-A-1、NCI-H1299细胞进行后续实验。FISH实验结果显示，THAP7-AS1位于SPC-A-1、NCI-H1299细胞的细胞质与细胞核中。见图1、2。

**图1:**
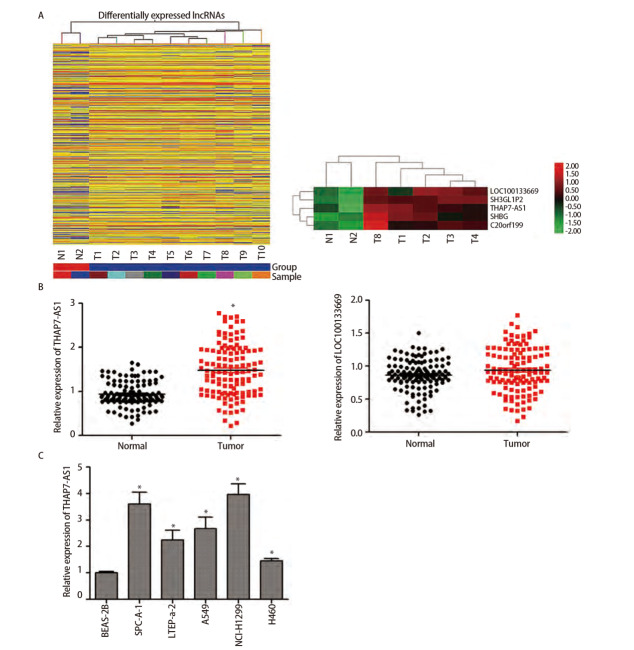
THAP7-AS1在肺癌中表达上调。A：10例肺癌与2例癌旁组织lncRNA表达谱聚类热图；B：120对肺癌与癌旁组织qRT-PCR检测THAP7-AS1、LOC100133669表达水平；C：肺癌细胞系qRT-PCR检测THAP7-AS1表达水平。*P<0.05。

**图2:**
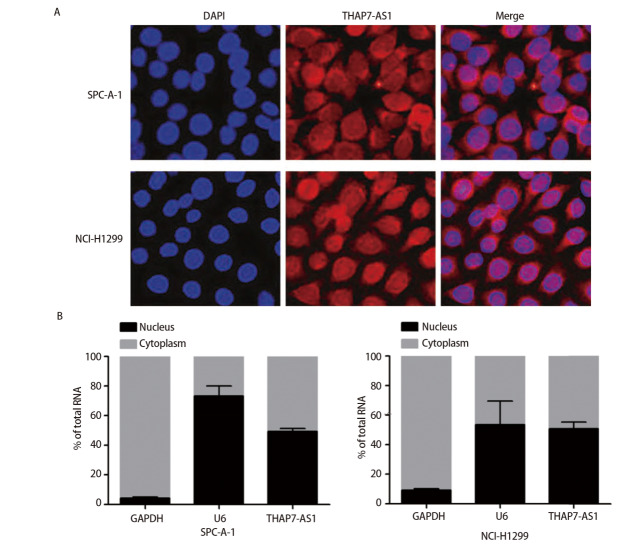
THAP7-AS1在SPC-A-1、NCI-H1299细胞中的定位（×400）。A：FISH实验染色结果；B：THAP7-AS1在SPC-A-1、NCI-H1299细胞的胞核和胞浆中的相对表达水平。

### 2.2 THAP7-AS1与肺癌诊断、预后及临床病理特征的关系

根据120对肺癌与癌旁组织THAP7-AS1表达水平评价THAP7-AS1对肺癌的诊断价值，绘制ROC曲线，其AUC值为0.737（95%CI: 0.608-0.847, P<0.05），提示THAP7-AS1对肺癌具有一定的诊断价值。Kaplan-Meier Plotter数据库生存分析结果显示，THAP7-AS1高表达的肺癌患者总生存率较低（P<0.05）。以THAP7-AS1表达的平均值将肺癌患者分为低表达组与高表达组，年龄、性别、吸烟、组织学类型、远处转移、分化程度与肺癌组织THAP7-AS1表达水平差异无统计学意义（P>0.05），肿瘤大小、肿瘤原发灶-淋巴结-转移（tumor-node-metastasis, TNM）分期、淋巴结转移与肺癌组织THAP7-AS1表达水平差异有统计学意义（P<0.05）。见图3、表2。

**图3:**
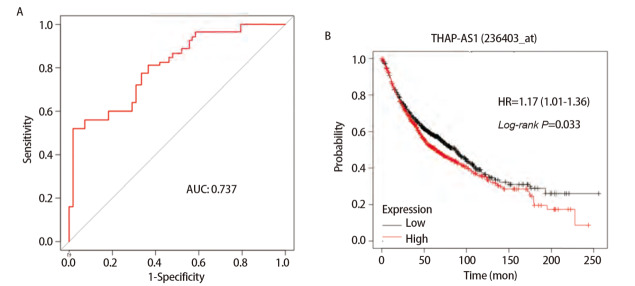
THAP7-AS1与肺癌诊断、预后及临床病理特征的关系。A：ROC曲线评价THAP7-AS1对肺癌的诊断价值；B：Kaplan-Meier Plotter数据库不同THAP7-AS1水平患者生存分析结果。

**表2:** THAP7-AS1表达水平与肺癌患者临床病理特征间的关系

Characteristics	n	THAP7-AS1		χ^2^	P
		High (n=60)	Low (n=60)		
Age, yr, n (%)				2.707	0.100
≥60	57 (47.50)	24 (40.00)	33 (55.00)		
<60	63 (52.50)	36 (60.00)	27 (45.00)		
Gender, n (%)				1.222	0.269
Male	68 (56.67)	31 (51.67)	37 (61.67)		
Female	52 (43.33)	29 (48.33)	23 (38.33)		
Smoker, n (%)				1.212	0.271
Yes	54 (45.00)	30 (50.00)	24 (40.00)		
No	66 (55.00)	30 (50.00)	36 (60.00)		
Histology, n (%)				1.310	0.519
Adenocarcinoma	68 (56.67)	31 (51.67)	37 (61.67)		
Squamous carcinoma	42 (35.00)	23 (38.33)	19 (31.67)		
Other type	10 (8.33)	6 (10.00)	4 (6.66)		
Tumor size, cm, n(%)				5.673	0.017
≥3	55 (45.83)	34 (56.67）	21 (35.00)		
<3	65 (54.17)	26 (43.33）	39 (65.00)		
TNM stage, n (%)				5.763	0.016
I-II	51 (42.50)	19 (31.67）	32 (53.33)		
III-IV	69 (57.50)	41 (68.33）	28 (46.67)		
Lymph node metastasis, n (%)				7.033	0.008
Yes	44 (36.67)	29 (48.33)	15 (25.00)		
No	76 (63.33)	31 (51.67)	45 (75.00)		
Distant metastasis,n (%)				0.352	0.553
Yes	37 (30.84)	20 (33.33)	17 (28.33)		
No	83 (69.16)	40 (66.67)	43 (71.67)		
Differentiation, n(%)				2.936	0.230
High	5 (4.17)	1 (1.67)	4 (6.66)		
Moderate	58 (48.33)	27 (45.00)	31 (51.67)		
Poor	57 (47.50)	32 (53.33)	25 (41.67)		

TNM: tumor-node-metastasis.

### 2.3 METTL3介导m6A修饰增强THAP7-AS1表达

使用SRAMP数据库预测THAP7-AS1序列中的m6A修饰位点，共发现7个位点。meRIP、qRT-PCR检测结果显示，THAP7-AS1中存在m6A修饰，过表达或下调METTL3可显著提高或抑制SPC-A-1、NCI-H1299细胞中THAP7-AS1的表达水平，肺癌组织中METTL3水平较癌旁组织显著上调（P<0.05），THAP7-AS1表达水平与METTL3表达水平呈正相关（r=0.4639, P<0.0001）。见图4。

**图4:**
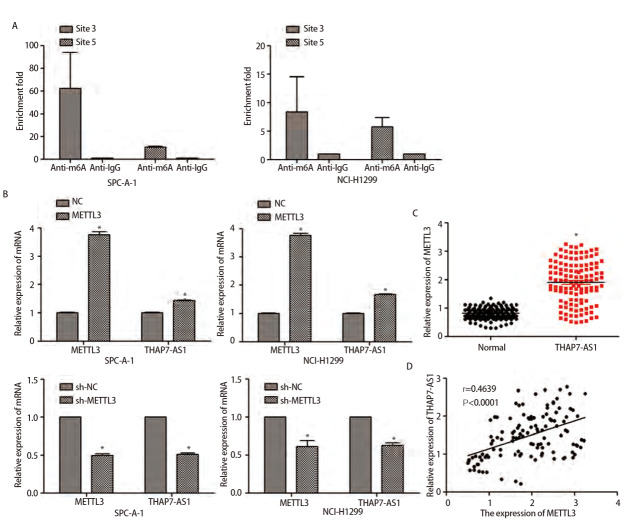
METTL3介导m6A修饰增强THAP7-AS1表达。A：m6A修饰的THAP7-AS1位点3、5在SPC-A-1、NCI-H1299细胞中富集；B：qRT-PCR检测过表达或下调METTL3对SPC-A-1、NCI-H1299细胞THAP7-AS1表达水平变化；C：qRT-PCR检测120对肺癌与癌旁组织THAP7-AS1表达情况；D：肺癌组织THAP7-AS1与METTL3表达水平的相关性分析。*P<0.05。

### 2.4 THAP7-AS1在体内外促进肺癌细胞的增殖、迁移和侵袭

MTS、EDU、克隆形成、划痕、Transwell实验结果显示，与SPC-A-1、NCI-H1299细胞NC组、sh-NC组相比，THAP7-AS1组增殖、克隆形成、迁移、侵袭能力提高（P<0.05），sh-THAP7-AS1组增殖、克隆形成、迁移、侵袭能力下降（P<0.05）。体内异种移植实验结果显示，与SPC-A-1、NCI-H1299细胞NC组相比，THAP7-AS1组肿瘤生长速度提升，体积、质量增大（P<0.05）。见图5-7。

**图5:**
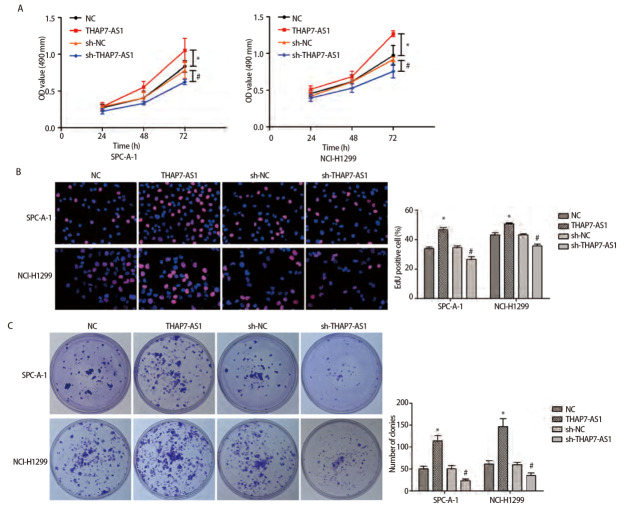
THAP7-AS1促进肺癌细胞增殖。A：构建SPC-A-1、NCI-H1299 THAP7-AS1过表达与沉默稳定表达细胞株，MTS检测细胞增殖率；B：EdU实验检测细胞DNA复制活性（×200）；C：克隆形成实验检测细胞克隆形成能力。*：与NC组相比，P<0.05；^#^：与sh-NC组相比，P<0.05。

**图6:**
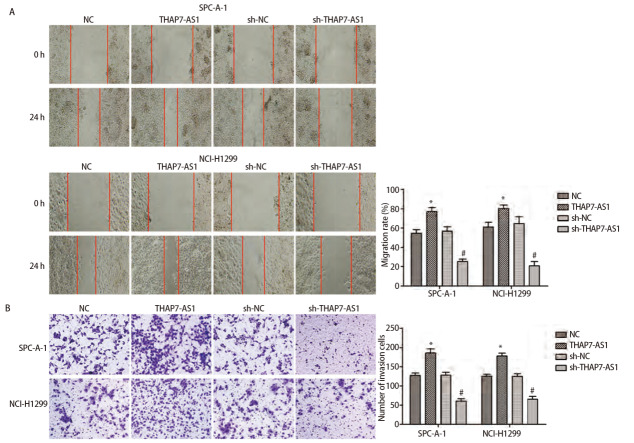
THAP7-AS1促进肺癌细胞迁移和侵袭。A：构建SPC-A-1、NCI-H1299 THAP7-AS1过表达与沉默稳定表达细胞株，划痕实验检测细胞迁移能力（×100）；B：Transwell实验检测细胞侵袭能力（×200）。*：与NC组相比，P<0.05；^#^：与sh-NC组相比，P<0.05。

**图7:**
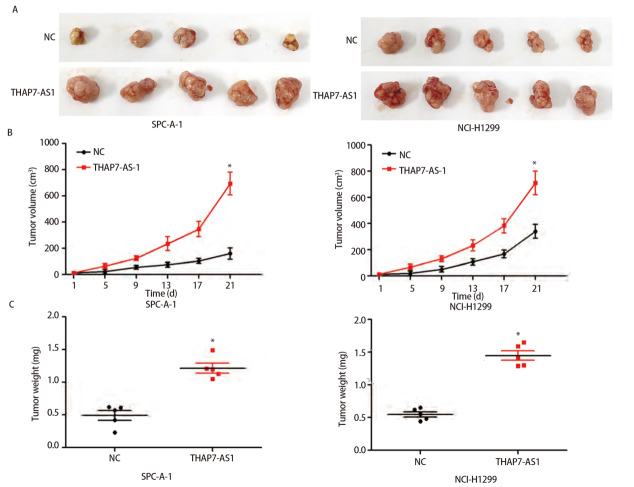
THAP7-AS1增强肺癌细胞成瘤能力。A：构建稳定过表达THAP7-AS1的SPC-A-1、NCI-H1299细胞株，体内异种移植实验肿瘤图片；B：移植瘤体积检测结果；C：移植瘤质量检测结果。*P<0.05。

### 2.5 THAP7-AS1结合CUL4B启动PI3K/AKT信号通路促进肺癌细胞增殖、迁移、侵袭

通过RNA下拉实验与质谱分析鉴定THAP7-AS1的蛋白质复合体，根据相对分子质量约为100 kDa、肽评分>100、相关研究显示参与肿瘤进展三个条件筛选出2个潜在的结合蛋白：CUL4B和STAT3。蛋白质组学分析、RIP检测结果显示，CUL4B与THAP7-AS1存在特异结合。MTS、划痕、Transwell实验结果显示，与SPC-A-1、NCI-H1299细胞Vector组相比，THAP7-AS1组增殖、迁移、侵袭能力升高（P<0.05）。Western blot检测结果显示，与SPC-A-1、NCI-H1299细胞Vector组相比，THAP7-AS1组PI3KCA、PI3KCD、p-PI3K、p-AKT、p-mTOR表达水平升高（P<0.05）。见图8-10。

**图8:**
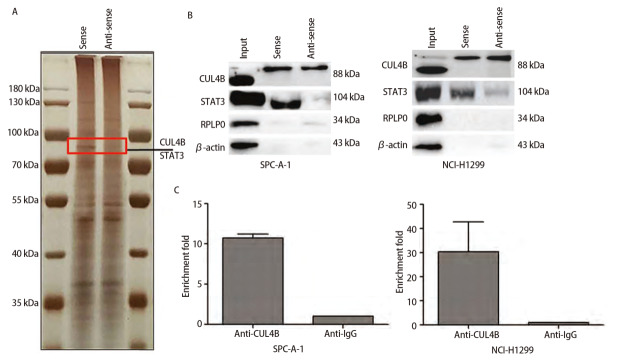
THAP7-AS1特异性结合蛋白筛选验证。A：THAP7-AS1蛋白质复合体银染实验结果；B：蛋白质组学Western blot验证结果；C：RIP检测CUL4B与THAP7-AS1结合情况。

**图9:**
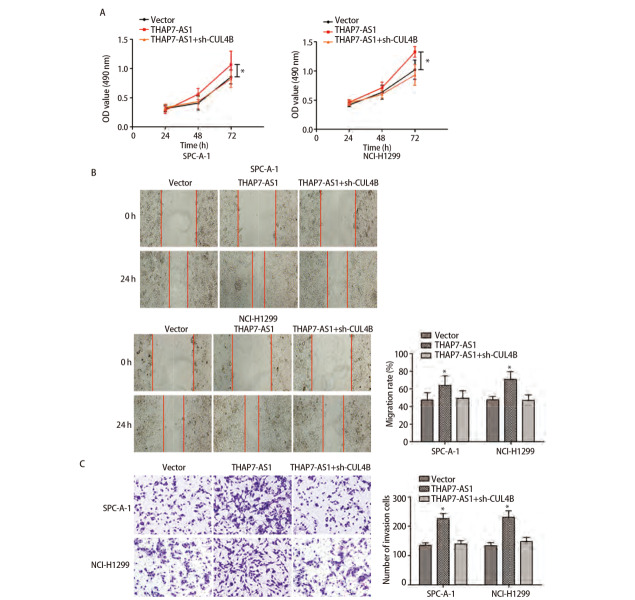
THAP7-AS1结合CUL4B促进肺癌细胞增殖、迁移、侵袭。A：构建SPC-A-1、NCI-H1299 THAP7-AS1过表达与沉默CUL4B稳定表达细胞株，MTS检测细胞增殖率；B：划痕实验检测细胞迁移能力（×100）；C：Transwell实验检测细胞侵袭能力（×200）。*P<0.05。

**图10:**
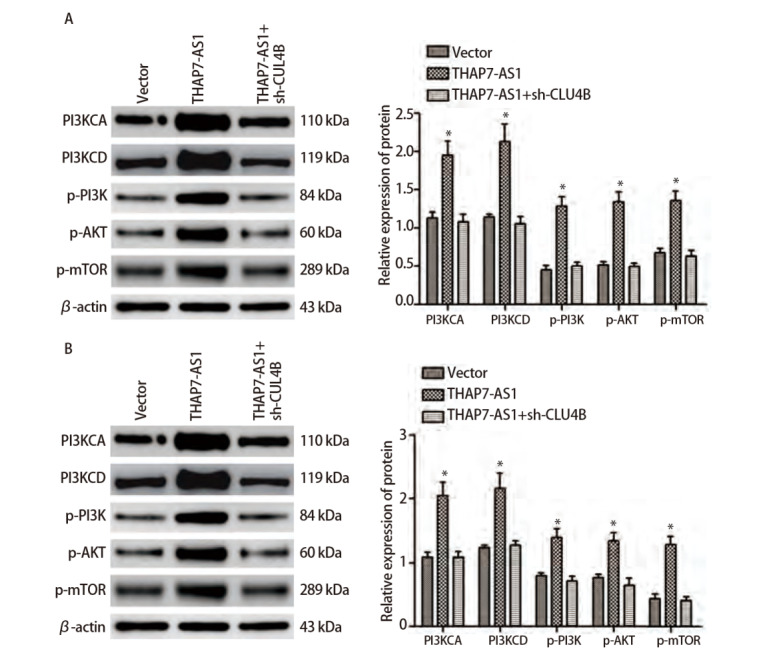
THAP7-AS1结合CUL4B启动PI3K/AKT信号通路。SPC-A-1（A）、NCI-H1299（B）THAP7-AS1过表达与沉默CUL4B稳定表达细胞株，Western blot检测PI3K/AKT信号通路相关蛋白表达情况。*P<0.05。

**图11:**
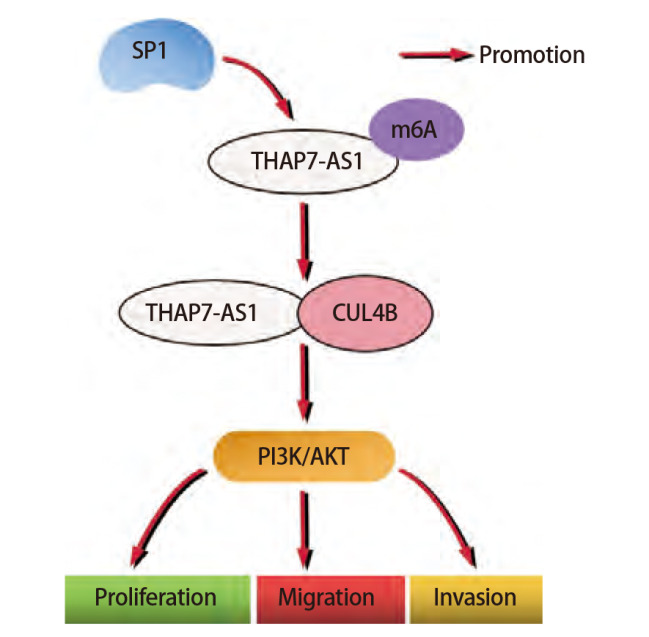
METTL3介导m6A修饰lncRNA THAP7-AS1表达上调促进肺癌发生的具体作用机制

### 2.6 METTL3介导m6A修饰lncRNA THAP7-AS1表达上调促进肺癌发生的具体作用机制

综合本研究结果发现，lncRNA THAP7-AS1在肺癌组织与细胞系中表达升高，对肺癌具有一定的诊断价值，其表达水平与患者总生存率、肿瘤大小、TNM分期、淋巴结转移相关。THAP7-AS1被SP1转录激活，经METTL3介导m6A修饰后稳定表达，通过与CUL4B结合激活PI3K/AKT信号通路促进SPC-A-1、NCI-H1299细胞增殖、迁移、侵袭，其可能的作用机制见图11。

## 3 讨论

研究^[14]^表明，多种lncRNA参与调节肺癌病理生理过程。LncRNA PKMYT1AR/miR-485-5p/PKMYT1轴通过抑制β-TrCP1介导的β-catenin蛋白的泛素化降解，促进非小细胞肺癌中肿瘤干细胞的维持，进而促进肿瘤发展^[15]^。LncRNA ABHD11‐AS1存在m6A修饰位点，METTL3介导m6A修饰诱导lncRNA ABHD11‐AS1增强NCI-H1299、NCI-H1650细胞增殖能力与瓦博格效应^[16]^。Liu等^[12]^的研究表明，lncRNA THAP7-AS1由特异性蛋白1激活表达，并由METTL3通过“阅读器”蛋白胰岛素样生长因子II mRNA结合蛋白1依赖的途径介导m6A修饰实现转录后稳定，激活下游PI3K/AKT信号通路促进胃癌发展。本研究通过lncRNA微阵列分析筛选肺癌组织与正常肺组织中差异表达的lncRNA，结果显示，lncRNA THAP7-AS1在肺癌组织中呈高表达。ROC曲线提示lncRNA THAP7-AS1对肺癌具有一定的诊断价值，其表达水平与患者总生存率、肿瘤大小、TNM分期、淋巴结转移有关，推测lncRNA THAP7-AS1的异常表达能够影响肺癌发展。METTL3在催化m6A修饰过程中占据主导地位，其水平变化可影响m6A修饰RNA甲基化进程^[17]^。结合SRAMP数据库预测分析结果、meRIP、qRT-PCR检测结果发现，THAP7-AS1存在m6A修饰，过表达或下调METTL3可影响SPC-A-1、NCI-H1299细胞THAP7-AS1表达，且METTL3在肺癌组织中表达上调，其水平与THAP7-AS1表达水平呈正相关，推测METTL3介导m6A修饰增强THAP7-AS1表达。

为了进一步探究THAP7-AS1在肺癌细胞中的生物学功能，本研究建立稳定过表达与沉默THAP7-AS1的SPC-A-1、NCI-H1299细胞株。各项细胞功能学实验结果显示，过表达THAP7-AS1增强了SPC-A-1、NCI-H1299细胞增殖、迁移、侵袭能力，而降低THAP7-AS1表达后，SPC-A-1、NCI-H1299细胞恶性生物学行为明显受到抑制；体内异种移植实验结果显示，过表达THAP7-AS1增强了SPC-A-1、NCI-H1299细胞的成瘤能力，推测THAP7-AS1对肺癌细胞SPC-A-1、NCI-H1299生长、迁移具有促进作用。LncRNA通过与蛋白质相互作用参与分子调控，推测THAP7-AS1可能与某些蛋白存在相互作用，调节肺癌细胞的恶性表型。经RNA下拉实验、质谱分析、蛋白质组学分析与RIP检测实验验证，THAP7-AS1与CUL4B存在特异结合。CUL4B是Cullin 4B-RING E3连接酶复合体中的一种支架蛋白，通过功能性核定位信号及其与核输入受体蛋白的相互作用在细胞核内积聚，在多种癌症中表达上调，发挥致癌作用^[18,19]^。MTS、划痕、Transwell实验结果显示，过表达THAP7-AS1的同时抑制CUL4B表达可降低THAP7-AS1对SPC-A-1、NCI-H1299细胞增殖、迁移、侵袭能力的促进效果。PI3K/AKT信号通路参与多种恶性肿瘤细胞增殖、血管生成、转移、耐药等方面，其相关分子在肺癌中异常表达^[20,21]^。Western blot检测结果显示，SPC-A-1、NCI-H1299细胞THAP7-AS1组PI3KCA、PI3KCD、p-PI3K、p-AKT、p-mTOR蛋白表达水平较Vector组升高，而THAP7-AS1+sh-CUL4B组蛋白表达水平与Vector组无明显差异，推测THAP7-AS1与CUL4B结合激活PI3K/AKT信号通路促进SPC-A-1、NCI-H1299细胞生长、迁移。

综上所述，lncRNA THAP7-AS1在肺癌中高表达，对肺癌具有一定的诊断价值，其表达水平与患者总生存率、不良临床病理特征有关，通过METTL3介导的m6A修饰稳定表达，与CUL4B结合激活PI3K/AKT信号通路，进而促进肺癌发生发展，具有成为肺癌新的生物标志物与治疗靶点的价值。

**Competing interests**

The authors declare that they have no competing interests.

**Author contributions**

Zhang Y and Wang YH conceived and designed the study. Zhang Y, Wang YH and Liu M performed the experiments. Wang YH and Liu M analyzed the data. Liu M contributed analysis tools. Zhang Y, Wang YH and Liu M provided critical inputs on design, analysis, and interpretation of the study. All the authors had access to the data. All authors read and approved the final manuscript as submitted.
